# Modeling Granulomas in Response to Infection in the Lung

**DOI:** 10.1371/journal.pone.0148738

**Published:** 2016-03-17

**Authors:** Wenrui Hao, Larry S. Schlesinger, Avner Friedman

**Affiliations:** 1 Mathematical Biosciences Institute, The Ohio State University, Columbus, OH, United States of America; 2 Center for Microbial Interface Biology & Department of Microbial Infection and Immunity, The Ohio State University, Columbus, OH, United States of America; 3 Mathematical Biosciences Institute & Department of Mathematics, The Ohio State University, Columbus, OH, United States of America; Fundació Institut d’Investigació en Ciències de la Salut Germans Trias i Pujol, Universitat Autònoma de Barcelona, SPAIN

## Abstract

Alveolar macrophages play a large role in the innate immune response of the lung. However, when these highly immune-regulatory cells are unable to eradicate pathogens, the adaptive immune system, which includes activated macrophages and lymphocytes, particularly T cells, is called upon to control the pathogens. This collection of immune cells surrounds, isolates and quarantines the pathogen, forming a small tissue structure called a granuloma for intracellular pathogens like *Mycobacterium tuberculosis* (Mtb). In the present work we develop a mathematical model of the dynamics of a granuloma by a system of partial differential equations. The ‘strength’ of the adaptive immune response to infection in the lung is represented by a parameter *α*, the flux rate by which T cells and M1 macrophages that immigrated from the lymph nodes enter into the granuloma through its boundary. The parameter *α* is negatively correlated with the ‘switching time’, namely, the time it takes for the number of M1 type macrophages to surpass the number of infected, M2 type alveolar macrophages. Simulations of the model show that as *α* increases the radius of the granuloma and bacterial load in the granuloma both decrease. The model is used to determine the efficacy of potential host-directed therapies in terms of the parameter *α*, suggesting that, with fixed dosing level, an infected individual with a stronger immune response will receive greater benefits in terms of reducing the bacterial load.

## Introduction

The lung encounters frequent challenges from inhaled particulate and microbes, the latter include intracellular pathogens of macrophages such as *M. tuberculosis (Mtb)*. Although this organ must effectively combat these invasions, it must do so without causing excessive inflammation. Alveolar macrophages play a large role in the innate immune response of the lung. However, when these unique, immune regulatory cells are unable to eradicate pathogens, the adaptive immune system, which includes activated macrophages and lymphocytes, particularly T cells, is called upon to control the pathogens. This collection of immune cells surrounds, isolates and quarantines the pathogen, forming a small tissue structure called a granuloma. Granulomas are well-organized structures with immune cells at various stages of differentiation [1]. Granulomas form shortly after infection [1]. Once formed, granulomas evolve [2]. In the present work, we develop a mathematical model of granuloma evolution in the context of tuberculosis (TB).

TB is an infectious disease caused by inhalation of Mtb. The bacteria disseminate inside and outside of the lung during infection, and form granulomas in various organs of the body [3]. Granulomas are the histologic hallmark of infection with Mtb. It is estimated that one third of the human population is infected with Mtb, predominantly in latent state. Only 10% of those infected with Mtb develop active TB disease over their lifetime, while 90% of otherwise healthy humans remain in a latent state following infection [4, 5]; however the percentage of those with latent infection that develop active disease increases if they are immunocompromised, e.g., HIV or diabetes [1]. Inhaled bacteria that enter the lung airway and are ingested by alveolar macrophages which are highly regulated in their immune responses, and their ability to destroy digested bacteria is limited. Alveolar macrophages as well as dendritic cells (DCs) that ingest bacteria migrate to the thoracic lymph nodes where T cells are primed. T cells that arrive at infected regions of the tissue prime macrophages to become classically activated, which helps control the bacteria. Interaction between the immune cells (macrophages, DCs and T cells) and bacteria results in granuloma formation [6]. A granuloma varies in size and activity during the course of latent and active TB, and may disappear [2, 7, 8].

**Fig 1:**
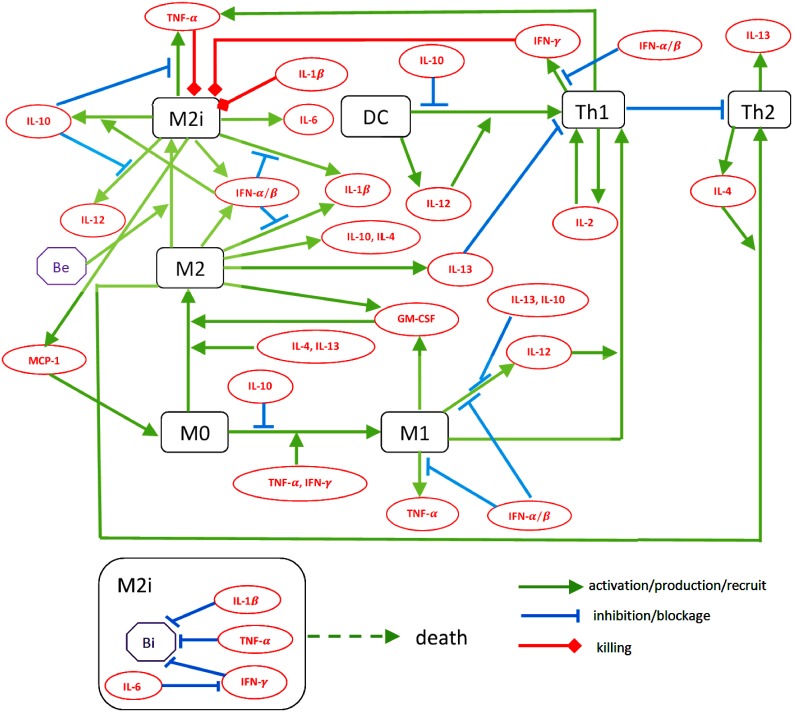
Schematic network d(See Table 1 for notation). Macrophage M0, from blood monocytes, polarize into either M1 or M2 macrophages under stimulation by different cytokines. Dendritic cells attract T cells from the lymph nodes into the granuloma. When an M2 macrophge phagocytoses extracellular bacteria Be, it becomes an infected macrophage M2i. M1, M2, M2i, D, Th1 and Th2 cells produce a variety of proinflammatory and anti-inflammatory cytokines, which upregulate or downregulate these cells. The growth of bacteria Bi inside macrophages M2i is inhibited by IL-1*β*, TNF-*α* and IFN-*γ* (modulated by IL-6). When an infected macrophage M2i bursts, its Bi are released to become extracellular bacteria.

In this paper we develop a spatial mathematical model of the granuloma. The model is based on the network shown in Fig 1. After infection with Mtb, alveolar macrophages secrete MCP-1 [9], which attracts monocytes from the blood migrate to the site of infection. The monocyte differentiate into macrophages M0 that are polarized to become either M2 macrophages under stimulation by IL-4, IL-13 or GM-CSF [10–12], or M1 macrophages under stimulation by TNF-*α* and IFN-*γ*, which is regulated by IL-10 [13]. M1 macrophages produce TNF-*α*, IL-12 and GM-CSF [14, 15]. M2 macrophages produce IL-1*β*[14], IL-10 [13], IL-13 [16] and IFN-*α*/*β*[14] while infected M2 macrophages (*M*_2*i*_) produce also TNF-*α*[14] but not IL-13. Dendritic cells play a critical role. After digesting external bacteria, Be, they travel to lymph nodes where they induce naive T cells to differentiate to either Th1 cells under IL-12 environment [17] or Th2 cells under IL-4 environment [18]. The newly formed Th1 cells become activated by contact with M1 macrophages and IL-12 [17], a process resisted by IL-10 [19], whereas Th2 cells become activated by contact with M2 macrophages and IL-4 [18], a process resisted by Th1 cells [18, 20, 21]. TNF-*α*, IL-1*β* and Th1-produced IFN-*γ* inhibits the growth of bacteria in infected macrophages *M*_2*i*_[13, 22]. On the other hand Th2 cells secrete IL-4 and IL-13 [11, 23] which enhance the polarization of M0 toward M2 [11, 12]. Th1 cells inhibit Th2 cells [18].

Neutrophils are typically first responders in host defence, but reports on their mycobactericidal capacity in tissue are conflicting, perhaps because of their inherent variability and short life span [24, 25]; we shall therefore not include them in the model.

More details on the various links in the network of Fig 1 will be given in the section “Mathematical model”.

The signaling network within a granuloma reflects the number of bacteria in the granuloma, the ability to grow within macrophages, and the more complex efforts of the immune system to kill the bacteria while keeping inflammation under control. Thus resident infected macrophages, as well as M1 and M2 macrophages, produce all classes of cytokines discussed above, but vary in the quantity of each, creating differential balancing of the cytokines and associated signaling networks. The “pro-inflammatory” versus “anti-inflammatory” response is also seen within T cell activities. Th1 cells produce IFN-*γ* and TNF-*α*, as well as IL-2, while Th2 cells produce IL-4 and IL-13; these two populations of T cells are mutually inhibiting.

Our mathematical model focuses on the evolution of a granuloma, once formed; we do not address the early mechanisms involved in granuloma formation. The evolution of the granuloma is modeled by a system of partial differential equations (PDEs) that takes place within the granuloma, and the boundary of the granuloma varies in time. Several mathematical models of TB granulomas represented by PDEs were developed and studied in [19, 26]. These models include several populations of macrophages classified according to their capacity to phagocytose and/or kill bacteria. The immigration of immune cells from the thoracic lymph nodes into the granuloma is represented by flux conditions at the boundary of the granuloma. Simulations of the model show how the number of bacteria that enter the granuloma affects the growth of the structure. We also simulate the effect of introducing potential host-directed therapies on the growth of the granuloma. These in-silico experiments explore how potential treatments reduce the bacteria load and limit granuloma growth, which could have important implications for the treatment of TB.

The correlation between granulomas in nonhuman primates and granulomas generated in silico was studied in [27] The in silico granuloma was derived by a mathematical model that describes, in terms of ODEs, the interaction between bacteria, immune cells, and the cytokines they secrete [27–29]. The concept of M1/M2 polarization ratio, *R*_*MP*_, was introduced in [30] as the ratio of pro-inflammatory signals (STAT1_*R*_, NF-*κB*_*R*_) to anti-inflammatory signals (STAT3_*R*_). This ratio was assumed to be linked to the immune response to infection with Mtb. A different approach to gauge the immune response is the “switching time”, which is the time (from initial infection) that it takes for M1 macrophages to exceed the number of infected M2 alveolar macrophages. This concept was introduced in [13] and used also in [27]. As shown in [13], a switching time of 55 days, for example, can be reduced to 34 days by injection of IFN-*γ*, which strengthens the immune response. The mathematical model in [13] uses a one compartment ODE model to describe the infection among bacteria, immune cells and the cytokines they produce, whereas the model in [27] accounts, in more detail, for the T cell response by using a two compartment model, one in the lung and the other for lymph nodes, both described by systems of ODEs.

In the present paper, we describe the evolution of granulomas by a system of PDEs, with bacteria, immune cells and cytokines that vary in time and space within the granuloma; as does the granuloma volume. Importantly, the immune response is represented phenomenologically in boundary conditions where a parameter *α* quantifies the rate by which macrophages and T cells enter into the granuloma; these ‘flux’ boundary conditions may be viewed as the contribution to the immune response from the lymph node tissue compartment. It has been shown in previous models ([13, 26, 27, 31] and the references therein) that recruitment of immune cells to the lung is a key factor in granuloma outcome. Herein we express this recruitment by a parameter *α*. The parameter *α* plays a critical role in our paper, in gauging the strength of the immune system. Indeed, there is a negative correlation between the flux rate *α* and the switching time: As *α* increases the switching time decreases. The biologically relevant values of *α* can then be determined by the fact that the switching time is typically from a few weeks to two months [13].

Therapeutic approaches to TB have been considered in [28, 29, 32–34] using both in silico experiments and nonhuman primate experiments. In these papers multi-scale and dynamical system approaches were developed and correlated with in vivo experiments; in particular, an anti IL-10 drug was considered. In the present paper, we use the PDE model to evaluate the efficacy of anti IL-10 and anti IL-13. As seen in Fig 1, IL-10 blocks an effective anti-TB response by the immune system. Indeed, it blocks polarization of M0 into M1 macrophages and, consequently, the production of IL-12 and TNF-*α* and the activation of Th1 cells. IL-10 has therefore been proposed to be a good target for TB therapy. Computational models for TB treatment with IL-10 Ab were already developed in [28, 32, 33] showing agreement with nonhuman primate experiments. We assume that IL-10 Ab is administered continuously (daily) for a period of 30 days, and assume, for illustration, that its effect is to reduce the production of the cytokine by 90%. We then proceed to consider with our model the following questions, applicable to personalized medicine: Does the reduction in granuloma volume depend on the immune response (i.e., on the flux rate parameter *α*) of the infected person, and does the reduction in granuloma volume depend on the time (relative to initial infection) when the treatment began? The answers to these questions will help determine the optimal therapeutic window for the infected individual. As seen from Fig 1, IL-13 enhances the polarization of M0 to M2 macrophages and it inhibits the activation of Th1 cells. Hence, like IL-10, IL-13 compromises the immune response to TB. We use our model, as in the case of IL-10, to explore the efficacy of treatment with IL-13 Ab.

## Mathematical model

The mathematical model is based on the diagram shown in Fig 1. The variables of the model are listed in Table 1.

**Table 1:** The variables of the model; concentration and densities are in units of *g*/*cm*^3^.

*M*_0_:	Interstitial macrophage density	*M*_1_:	M1 macrophage density
*M*_2_:	M2 macrophage density	*M*_2*i*_:	Infected M2 macrophage density
*T*_1_:	Th1 cell density	*T*_2_:	Th2 cell density
*I*_2_:	IL-2 concentration	*I*_10_:	IL-10 concentration
*I*_12_:	IL-12 concentration	*I*_13_:	IL-13 concentration
*T*_*α*_:	TNF-*α* concentration	*I*_*γ*_:	IFN-*γ* concentration
*G*:	GM-CSF concentration	**u**:	cell velocity
*D*:	dendritic cell density	*I*_*α*_	IFN-*α* and *β*
*T*_0_:	naive CD4^+^ T cells density	*D*_0_	Interstitial dendritic cells density
*B*_*e*_:	extracellular bacteria	*B*_*i*_	intercellular bacteria
*I*_1*β*_:	IL-1*β* concentration		

### Equations for macrophages (*M*_1_, *M*_2_)

We assume that there is a constant source of macrophages (*M*_0_) migrating into the airspace and then differentiating into either alternatively (M2) activated macrophages or into activated M1 macrophages, as they enter the alveolar space [35] depending on cues they receive from several cytokines. The equations for macrophages are given as follows:

$$\begin{array}{ccc} \frac{\partial M_{1}}{\partial t}+\nabla \cdot \left(uM_{1}\right)-D_{M}\Delta M_{1} & = & \underset{chemotaxis}{\underset{︸}{-\nabla (M_{1}\chi_{C}\nabla P)}}+\underset{M_{0}\to M_{1}}{\underset{︸}{\lambda_{M_{0}}\frac{\varepsilon_{1}}{\varepsilon_{1}+\varepsilon_{2}}M_{0}}}+\underset{M_{2}\to M_{1}}{\underset{︸}{\lambda_{M_{1}}\frac{\varepsilon_{1}}{\varepsilon_{1}+\varepsilon_{2}}M_{2}}}\underset{death}{\underset{︸}{-d_{M_{1}}M_{1}}}, \end{array}$$ (1)

$$\begin{array}{ccc} \frac{\partial M_{2}}{\partial t}+\nabla \cdot \left(uM_{2}\right)-D_{M}\Delta M_{2} & = & \underset{personalized\;source}{\underset{︸}{A}}+\underset{M_{0}\to M_{2}}{\underset{︸}{\lambda_{M_{0}}\frac{\varepsilon_{2}}{\varepsilon_{1}+\varepsilon_{2}}M_{0}}}\underset{M_{2}\to M_{1}}{\underset{︸}{-\lambda_{M_{1}}\frac{\varepsilon_{1}}{\varepsilon_{1}+\varepsilon_{2}}M_{2}}}\\ & & \underset{M_{2}\to M_{2i}}{\underset{︸}{-\lambda_{M_{2i}}M_{2}\frac{Be}{Be+K_{Be}}}}\underset{death}{\underset{︸}{-d_{M_{2}}M_{2}}}, \end{array}$$ (2)

$$\begin{array}{ccc} \frac{\partial M_{2i}}{\partial t}+\nabla \cdot \left(uM_{2i}\right)-D_{M}\Delta M_{2i} & = & \underset{M_{2}\to M_{2i}}{\underset{︸}{\lambda_{M_{2i}}M_{2}\frac{Be}{Be+K_{Be}}}}\underset{B_{i}\to B_{e}}{\underset{︸}{-\lambda_{Be}M_{2i}\frac{B_{i}^{2}}{B_{i}^{2}+{\left(NM_{2i}\right)}^{2}+\delta }}}\underset{death}{\underset{︸}{-d_{M_{2i}}M_{2i}}}, \end{array}$$ (3)

where

$$\begin{array}{ccc} \varepsilon_{1} & = & \left(\lambda_{MI_{\gamma }}\frac{I_{\gamma }}{I_{\gamma }+K_{I_{\gamma }}}+\lambda_{MT_{\alpha }}\frac{T_{\alpha }}{T_{\alpha }+K_{T_{\alpha }}}\right)\frac{1}{1+I_{10}/K_{I_{10}}}, \end{array}$$

$$\begin{array}{ccc} \varepsilon_{2} & = & \lambda_{MG}\frac{G}{G+K_{G}}+\lambda_{MI_{4}}\frac{I_{4}}{I_{4}+K_{I_{4}}}+\lambda_{MI_{13}}\frac{I_{13}}{I_{13}+K_{I_{13}}}. \end{array}$$

The function *ε*_1_ is increasing in *I*_*γ*_ and *T*_*α*_ and decreasing in *I*_10_, whereas the function *ε*_2_ is increasing in GM-CSF, *I*_4_ and *I*_13_. When interstitial macrophages enter the lung (from the blood vessels) they differentiate into either M1 or M2. *I*_*γ*_ and *T*_*α*_ increase the affinity to differentiate into M1 macrophages, and this is regulated by *I*_10_[13], whereas affinity toward M2 differentiation is provided by GM-CSF [10] and *I*_4_, *I*_13_[11, 12]. These facts are accounted for by the first terms on the right-hand side of Eqs (1) and (2). The second term in these equations represents the transition from M2 to M1 induced by *I*_*γ*_ and *T*_*α*_[13]. The third term in Eq (3) accounts for infection of M2 by external bacteria, and the second term on the right-hand side of Eq (3) represents the burst of internal bacteria as infected macrophages undergo necrosis [13]; *N* is the average number of bacteria at the time of necrosis. The last term in each of Eqs (1)–(3) is death by apoptosis.

### Equations for DCs (*D*)

Dendritic cells are activated by contact with infected macrophages, a process resisted by *I*_10_[13]. Hence

$$\begin{array}{ccc} \frac{\partial D}{\partial t}+\nabla \cdot \left(uD\right)-D_{D}\Delta D & = & \underset{\text{ingesting}\;\;\text{Be}}{\underset{︸}{\lambda_{D}D_{0}\frac{Be}{Be+K_{Be}}}}\underset{death}{\underset{︸}{-d_{D}D.}}\; \end{array}$$ (4)

### Equations for T cells (*T*_1_, *T*_2_)

Resting naive T cells in the thoracic lymphoid tissue are primed while in contact with antigen presented by DCs, and develop into an intermediate stage called Th0 cells, which we denote by *T*_0_, The Th0 cells migrate into the lung and are activated into Th1 by contact with M1 macrophages in an IL-12 environment [17], a process down-regulated by IL-10 [13], or into Th2 cells by contact with M2 macrophages under an IL-4 environment [18]. IL-2 induces proliferation of Th1 cells [15]. Both activation and proliferation of Th1 cells are antagonized by IL-13 [36], while the activation of Th2 cells is antagonized by Th1 [18, 20, 21]. Hence, the equation of Th1 and Th2 cells is given as follows:

$$\begin{array}{ccc} \frac{\partial T_{1}}{\partial t}+\nabla \cdot \left(uT_{1}\right)-D_{T}\Delta T_{1} & = & (\lambda_{T_{1}M_{1}}T_{0}\frac{M_{1}}{M_{1}+K_{M_{1}}}\frac{I_{12}}{I_{12}+K_{I_{12}}}\frac{1}{1+I_{10}/K_{I_{10}}}\\ & & +\lambda_{TI_{2}}\frac{I_{2}}{I_{2}+K_{I_{2}}}T_{1})\frac{1}{1+I_{13}/K_{I_{13}}}\underset{death}{\underset{︸}{-d_{T_{1}}T_{1}}}, \end{array}$$ (5)

$$\begin{array}{ccc} \frac{\partial T_{2}}{\partial t}+\nabla \cdot \left(uT_{2}\right)-D_{T}\Delta T_{2} & = & \underset{activation}{\underset{︸}{\lambda_{T_{2}}T_{0}\frac{M_{2}}{M_{2}+K_{M_{2}}}\frac{I_{4}}{I_{4}+K_{I_{4}}}\frac{1}{1+T_{1}/K_{T_{1}}}}}\underset{death}{\underset{︸}{-d_{T_{2}}T_{2}}}. \end{array}$$ (6)

### Equations for bacteria (*B*_*e*_, *B*_*i*_)

The extracellular and intercellular bacteria satisfy the following equations:

$$\begin{array}{ccc} \frac{\partial B_{e}}{\partial t}-D_{B_{e}}\Delta B_{e} & = & \underset{B_{i}\to B_{e}}{\underset{︸}{\lambda_{Be}NM_{2i}\frac{B_{i}^{2}}{B_{i}^{2}+{\left(NM_{2i}\right)}^{2}+\delta }}}\underset{\text{eating}\;\text{Be}}{\underset{︸}{-\left(\lambda_{M_{2}i}M_{2}+\lambda_{D}D_{0}\right)\frac{B_{e}}{B_{e}+K_{B_{e}}}}},\; \end{array}$$ (7)

$$\begin{array}{ccc} \frac{\partial B_{i}}{\partial t}-D_{B_{i}}\Delta B_{i} & = & \underset{B_{e}\to B_{i}}{\underset{︸}{\lambda_{M_{2}i}M_{2}\frac{B_{e}}{B_{e}+K_{B_{e}}}}}\underset{B_{i}\to B_{e}}{\underset{︸}{-\lambda_{Be}NM_{2i}\frac{B_{i}^{2}}{B_{i}^{2}+{\left(NM_{2i}\right)}^{2}+\delta }}}\underset{death}{\underset{︸}{-d_{M_{2}i}B_{i}}}\\ & \; & +\underset{growth\;of\;B_{i}}{\underset{︸}{\lambda_{Bi}\frac{B_{i}\left(1-\frac{B_{i}^{2}}{B_{i}^{2}+{\left(NM_{2i}\right)}^{2}+\delta }\right)}{\left(1+I_{\beta }/K_{I_{\beta }}\right)\left(1+I_{\gamma }/\left(K_{I_{\gamma }}+I_{6}\right)\right)\left(1+T_{\alpha }/K_{T_{\alpha }}\right)}}}.\;\; \end{array}$$ (8)

Extracellular bacteria emerge from bursting of infected macrophages, with *N* bacteria per macrophage, and they are phagocytosed by DCs and macrophages. The intracellular bacteria population increases when M2 macrophages phagocytose extracellular bacteria, but also by proliferation within infected macrophages: the proliferation is modeled by logistic growth with carrying capacity limited by the macrophage capacity to combat the bursting of cells, and this rate is inhibited by *I*_*γ*_, *T*_*α*_[13] and IL-1*β*[22]; inhibition by IFN-*γ* is modulated by IL-6 [14, 37].

### Equation for MCP-1

MCP-1 is produced by M2i macrophages [9]:

$$\begin{array}{ccc} \frac{\partial P}{\partial t}-D_{P}\Delta P & = & \underset{production}{\underset{︸}{\left(\lambda_{PM_{2}i}M_{2i}\right)}}\underset{degradation}{\underset{︸}{-d_{P}P}}. \end{array}$$ (9)

### Equation for Interleukins

IL-1*β* is produced by M2 and M2i macrophages and resisted by IFN-*α*[14, 38]:

$$\begin{array}{ccc} \frac{\partial I_{1\beta }}{\partial t}-D_{I_{1\beta }}\Delta I_{1\beta } & = & \underset{production}{\underset{︸}{\left(\lambda_{I_{1\beta }M_{2}}M_{2}+\lambda_{I_{1\beta }M_{2}i}M_{2i}\right)\frac{1}{1+I_{\alpha }/K_{I_{\alpha }}}}}\underset{degradation}{\underset{︸}{-d_{I_{1\beta }}I_{1\beta }}}. \end{array}$$ (10)

IL-2 is produced by Th1 cells [15]:

$$\begin{array}{ccc} \frac{\partial I_{2}}{\partial t}-D_{I_{2}}\Delta I_{2} & = & \underset{production}{\underset{︸}{\lambda_{I_{2}T_{1}}T_{1}}}\underset{degradation}{\underset{︸}{-d_{I_{2}}I_{2}}}. \end{array}$$ (11)

IL-4 is produced by Th2 cells and M2 macrophages [23, 39], hence

$$\begin{array}{ccc} \frac{\partial I_{4}}{\partial t}-D_{I_{4}}\Delta I_{4} & = & \underset{production}{\underset{︸}{\lambda_{I_{4}T_{2}}T_{2}+\lambda_{I_{4}M_{2}}M_{2}}}\underset{degradation}{\underset{︸}{-d_{I_{4}}I_{4}}}, \end{array}$$ (12)

IL-6 is produced by infected M2 macrophages [40, 41], hence

$$\begin{array}{ccc} \frac{\partial I_{6}}{\partial t}-D_{I_{6}}\Delta I_{6} & = & \underset{production}{\underset{︸}{\lambda_{I_{6}M_{2i}}M_{2i}}}\underset{degradation}{\underset{︸}{-d_{I_{6}}I_{6}}}, \end{array}$$ (13)

IL-10 is produced by M2 and M2i macrophages and by DCs [13]. IL-12 is produced by M1 and M2i macrophages and by DCs, a process resisted by IL-10 [13] and IL-13 [42]. Hence IL-10 and IL-12 satisfy the equations:

$$\begin{array}{ccc} \frac{\partial I_{10}}{\partial t}-D_{I_{10}}\Delta I_{10} & = & \underset{production}{\underset{︸}{\lambda_{I_{10}M_{2i}}M_{2i}+\lambda_{I_{10}D}D+\lambda_{I_{10}M_{2}}M_{2}}}\underset{degradation}{\underset{︸}{-d_{I_{10}}I_{10}}}, \end{array}$$ (14)

$$\begin{array}{ccc} \frac{\partial I_{12}}{\partial t}-D_{I_{12}}\Delta I_{12} & = & \underset{production}{\underset{︸}{\left(\lambda_{I_{12}M_{2i}}M_{2i}+\lambda_{I_{12}D}D+\lambda_{I_{12}M_{1}}M_{1}\right)\frac{1}{1+I_{10}/K_{I_{10}}}\frac{1}{1+I_{13}/K_{I_{13}}}}}\underset{degradation}{\underset{︸}{-d_{I_{12}}I_{12}.}} \end{array}$$ (15)

IL-13 is produced by alveolar macrophages (M2) [16] and (mostly) by Th2 cells [11], so that

$$\begin{array}{ccc} \frac{\partial I_{13}}{\partial t}-D_{I_{13}}\Delta I_{13} & = & \underset{production}{\underset{︸}{\lambda_{I_{13}T_{2}}T_{2}+\lambda_{I_{13}M}M_{2}}}\underset{degradation}{\underset{︸}{-d_{I_{13}}I_{13}}}, \end{array}$$ (16)

where *λ*_*I*_13_*T*_2__ is larger than *λ*_*I*_13_*M*_.

### Equation for TNF-*α*

TNF-*α* is produced by M2i and M1 macrophages, processes resisted by IL-10 [43] and IFN-*α*[14]:

$$\begin{array}{ccc} \frac{\partial T_{\alpha }}{\partial t}-D_{T_{\alpha }}\Delta T_{\alpha } & = & \underset{production}{\underset{︸}{\left(\lambda_{T_{\alpha }M_{2i}}M_{2i}+\lambda_{T_{\alpha }M_{1}}M_{1}\right)\frac{1}{\left(1+I_{10}/K_{I_{10}}\right)\left(1+I_{\alpha }/K_{I_{\alpha }}\right)}}}\underset{degradation}{\underset{︸}{-d_{T_{\alpha }}T_{\alpha }}}.\; \end{array}$$ (17)

### Equations for IFN

IFN-*α*/*β* is produced by M2i and M2 macrophages, a process resisted by IL-1*β*[14]:

$$\begin{array}{ccc} \frac{\partial I_{\alpha }}{\partial t}-D_{I_{\alpha }}\Delta I_{\alpha } & = & \underset{production}{\underset{︸}{\left(\lambda_{I_{\alpha }M_{2i}}M_{2i}+\lambda_{I_{\alpha }M_{2}}M_{2}\right)\frac{1}{1+I_{1\beta }/K_{I_{1\beta }}}}}\underset{degradation}{\underset{︸}{-d_{I_{\alpha }}I_{\alpha }}}, \end{array}$$ (18)

and IFN-*γ* is produced by Th1 [15], a process resisted by IFN-*α*[14],

$$\begin{array}{ccc} \frac{\partial I_{\gamma }}{\partial t}-D_{I_{\gamma }}\Delta I_{\gamma } & = & \underset{production}{\underset{︸}{\lambda_{I_{\gamma }T_{1}}T_{1}\frac{1}{1+I_{\alpha }/K_{I_{\alpha }}}}}\underset{degradation}{\underset{︸}{-d_{I_{\gamma }}I_{\gamma }}}. \end{array}$$ (19)

### Equation for GM-CSF (*G*)

GM-CSF is secreted by macrophages [15]; it satisfies the equation

$$\begin{array}{ccc} \frac{\partial G}{\partial t}-D_{G}\Delta G & = & \underset{production}{\underset{︸}{\lambda_{GM1}M_{1}+\lambda_{GM2}M_{2}}}\underset{degradation}{\underset{︸}{-d_{G}G}}. \end{array}$$ (20)

### Equation for *u*

We assume uniform density of the total cell population within the granuloma, so that

$$D+M_{1}+M_{2}+M_{2i}+T_{1}+T_{2}=\text{ const. }=\theta \text{,}$$ (21)

and take *θ* = 1 *g*/*cm*^3^[15].

We assume that the granuloma occupies a spherical region where the radius *R*(*t*) may change in time {*r* < *R*(*t*)} and assume no-flux boundary conditions at *r* = *R*(*t*). We take all the functions in Eqs (1)–(16) to be radially symmetric. By adding all the equations for the variables in Eq (17) and assuming that all their diffusion coefficients are equal, we get an equation for $\nabla \cdot u=\frac{1}{r^{2}}\frac{\partial }{\partial r}\left(r^{2}v\right)$, where *v* is the common radial cells velocity:

$$\begin{array}{c} \frac{1}{r^{2}}\frac{\partial }{\partial r}\left(r^{2}v\right)=\sum \;\text{Right-hand}\;\text{sides}\;\text{of}\;\text{(1)-(6)}. \end{array}$$ (22)

## Boundary conditions

The dynamics of free boundary is given by the kinetic equation

$$\begin{array}{c} \frac{dR(t)}{dt}=v\left(R\left(t\right),t\right). \end{array}$$ (23)

We assume that the source of migrating naive T cells from the lymph nodes, *T*_0_, contributes to Th1 and Th2 cells not only within the granuloma, as in Eqs (5) and (6), but also through the boundary conditions:

$$\begin{array}{c} \frac{\partial T_{1}}{\partial n}+\alpha_{T_{1}}\left(D,I_{12}\right)\left(T_{1}-T_{0}\right)=0,\;\frac{\partial T_{2}}{\partial n}+\alpha_{T_{2}}\left(D,I_{4}\right)\left(T_{2}-T_{0}\right)=0, \end{array}$$ (24)

where $\alpha_{T_{1}}\left(D,I_{12}\right)=\alpha \frac{D}{D+D_{0}}\frac{I_{12}}{I_{12}+K_{I_{12}}}$, and $\alpha_{T_{2}}\left(D,I_{4}\right)=\alpha \frac{D}{D+D_{0}}\frac{I_{4}}{I_{4}+K_{I_{4}}}$.

Similarly we assume that the source of migrating macrophages *M*_0_ from the lymph nodes also contributes to *M*_1_ macrophages through the boundary condition

$$\begin{array}{c} \frac{\partial M_{1}}{\partial n}+\alpha_{M}\left(I_{\gamma },T_{\alpha }\right)\left(M_{1}-M_{0}\right)=0, \end{array}$$ (25)

where $\alpha_{M}\left(I_{\gamma },T_{\alpha }\right)=\alpha \frac{D}{D+D_{0}}(\frac{I_{\gamma }}{I_{\gamma }+K_{I_{\gamma }}}+\frac{T_{\alpha }}{T_{\alpha }+K_{T_{\alpha }}})$.

We take

$$\begin{array}{c} \frac{\partial I_{4}}{\partial n}+\alpha_{0}I_{4}=0,\;\frac{\partial I_{12}}{\partial n}+\alpha_{0}I_{12}=0,\;\frac{\partial P}{\partial n}+\alpha_{0}P=0,\;\frac{\partial T_{\alpha }}{\partial n}+\alpha_{0}T_{\alpha }=0\;\text{and}\;\frac{\partial I_{\gamma }}{\partial n}+\alpha_{0}I_{\gamma }=0, \end{array}$$ (26)

where *α*_0_ > 0 since some of these cytokines are consumed at the boundary. Similarly, we assume that

$$\begin{array}{c} \frac{\partial D}{\partial n}+\alpha_{0}D=0 \end{array}$$ (27)

on the boundary. The dimension of the parameters *α*_0_, *α* is 1/day. We take *α*_0_ = 1, but *α* will vary depending on the strength of the immune system; *α* is viewed as a parameter that gauges the immune response. Finally since all other variables (including M2 and M2i) are not directly involved with the migration of immune cells from the lymph nodes, we assume no-flux boundary conditions:

$$\begin{array}{c} \frac{\partial X}{\partial n}=0,\;\text{for}\;\text{the}\;\text{remaining}\;\text{variables.} \end{array}$$ (28)

## Initial conditions

We estimate that in the initial phase of granuloma formation 80% of the cells are macrophages, and most of them are M2 macrophages; at this phase the number of T cells is small (only few have arrived from the lymph nodes [44]) and so is the number of activated DCs. Accordingly, we take the density of cells in units of *g*/*cm*^3^ as follows:

$$\begin{array}{c} M_{2}=0.8,\;M_{1}=0.05,M_{2i}=0.1,\;T_{1}=0.02,\;T_{2}=0\;\text{and}\;D=0.03. \end{array}$$ (29)

Because of presumed similarities in the granulomas produced in TB and sarcoidosis, at least in the early stages [45, 46], we assume the following concentration of cytokines in units of *g*/*cm*^3^[15]:

$$\begin{array}{ccc} & & P=8.56\times 10^{-7},\;I_{4}=1.7\times 10^{-8},\;I_{12}=2.49\times 10^{-6},\;I_{1}=2.47\times 10^{-8},\\ & & I_{2}=1.29\times 10^{-8},\;I_{6}=2.03\times 10^{-9},\;I_{10}=7.45\times 10^{-10},\;I_{13}=1.63\times 10^{-8},\\ & & T_{\alpha }=1.24\times 10^{-7},\;I_{\alpha }=8.53\times 10^{-9},\;I_{\gamma }=9.87\times 10^{-10},\;G=8.51\times 10^{-8}. \end{array}$$ (30)

We next assume that when the granuloma is formed, it occupies a sphere of radius 0.01 cm [47], that is, *R*(0) = 0.01 cm. The size of Mtb is 2–4 *μm* in length and 0.2–0.5 *μm* in width. Accordingly we take the mass of one Mtb to be 4 × 10^−13^ *g*. Then dif one Mtb in the granuloma dis replaced by a uniform mass, then the cocentration of this mass will be $\frac{4\times 10^{-13}\;g}{\frac{4}{3}\pi 10^{-6}\;cm^{3}}\approx 1\times 10^{-7}$ *g*/*cm*^3^. We assume that at the time when the granuloma was formed there were 10^2^ Be and 10^3^ Bi. Hence, initially,

$$\begin{array}{c} Be\left(0\right)=10^{-5}\;g/cm^{3}\;\text{and}\;Bi\left(0\right)=10^{-4}\;g/cm^{3}; \end{array}$$ (31)

the simulations, given below, do not change qualitatively if we use other initial conditions for the bacteria.

## Results

**Fig 2:**
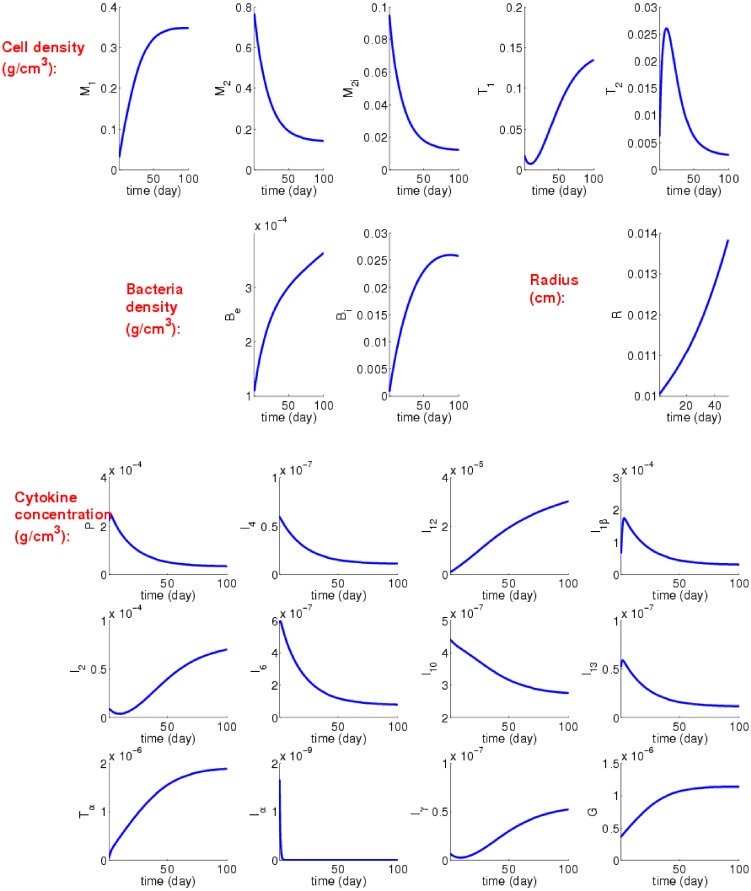
Simulation results over 100 days with *α* = 1 and initial inhalation of 10^2^ Be and 10^3^ Bi; and all of the units are in *g*/*cm*^3^. The symbols for cells and cytokines are listed in Table 1, and all the parameters are listed in Tables 2 and 3.

We first explore the PDE system Eqs (1)–(22), boundary conditions Eqs (23)–(28), initial conditions Eqs (29)–(31) and the parameters of Tables 2 and 3 when *α* = 1, where *α* is the parameter representing the immune strength. The profiles of all of the model’s variables in the first 100 days are shown in Fig 2.

**Table 2:** Parameters’ description and value.

Parameter	Description	Value
*D*_*M*_	diffusion coefficient of macrophage	8.64 × 10^−7^ *cm*^2^ day^−1^[15, 55, 56]
*D*_*D*_	diffusion coefficient of dendritic cell	8.64 × 10^−7^ *cm*^2^ day^−1^[15, 55, 56]
*D*_*T*_	diffusion coefficient of T cell	8.64 × 10^−7^ *cm*^2^ day^−1^[15, 55, 56]
*D*_*Be*_	diffusion coefficient of Be	1 × 10^−6^ *cm*^2^ day^−1^[15]
*D*_*Bi*_	diffusion coefficient of Bi	8.64 × 10^−7^ *cm*^2^ day^−1^[15]
*D*_*I*_*γ*__	diffusion coefficient of IFN-*γ*	1.08 × 10^−2^ *cm*^2^ day^−1^[15]
*D*_*T*_*α*__	diffusion coefficient of TNF-*α*	1.29 × 10^−2^ *cm*^2^ day^−1^[15]
*D*_*P*_	diffusion coefficient of MCP-1	1.78 × 10^−2^ *cm*^2^ day^−1^[15]
*D*_*I*_1*β*__	diffusion coefficient of IL-1*β*	1.08 × 10^−2^ *cm*^2^ day^−1^[15]
*D*_*I*_2__	diffusion coefficient of IL-2	1.08 × 10^−2^ *cm*^2^ day^−1^[15]
*D*_*I*_4__	diffusion coefficient of IL-4	1.08 × 10^−2^ *cm*^2^ day^−1^[15]
*D*_*I*_10__	diffusion coefficient of IL-10	1.08 × 10^−2^ *cm*^2^ day^−1^[15]
*D*_*I*_12__	diffusion coefficient of IL-12	1.08 × 10^−2^ *cm*^2^ day^−1^[15]
*D*_*I*_13__	diffusion coefficient of IL-13	1.08 × 10^−2^ *cm*^2^ day^−1^[15]
*D*_*G*_	diffusion coefficient of GM-CSF	1.728 × 10^−2^ *cm*^2^ day^−1^[15]
*D*_*T*_*α*__	diffusion coefficient for TNF-*α*	1.29 × 10^−2^ *cm*^2^ day^−1^[15]
*λ*_*M*_0__	differentiation rate of M0 to M1/M2	9.3 × 10^−3^/day estimated
*λ*_*M*_1__	maximal rate at which M2 is activated to become M1	6 × 10^−3^/day [13] & estimated
*λ*_*MI*_*r*__	production rate by IFN-*γ*	10^−3^/day estimated
*λ*_*MT*_*α*__	production rate by TNF-*α*	10^−3^/day estimated
*λ*_*MG*_	production rate by GM-CSF	10^−3^/day estimated
*λ*_*MI*_4__	production rate by IL-4	10^−3^/day estimated
*λ*_*MI*_13__	production rate by IL-13	10^−3^/day estimated
*λ*_*M*_2*i*__	maximal rate at which M2 phagocytose (extracellular) bacteria	0.28/day [13]
*λ*_*D*_	production rate of DCs	0.06/day [13]
*λ*_*Be*_	burst rate of *M*_2*i*_	1/day [13]
*λ*_*T*1*M*1_	production rate of Th1 cells by M1 macrophages and IL-12	0.23/day [13] & estimated
*λ*_*TI*_2__	production rate of Th1 cells by IL-2	1/day [15]
*λ*_*T*2_	production rate of Th2 cells	0.8/day [15]
*λ*_*I*_*γ*_*T*_1__	production rate of IFN-*γ* by Th1 cells	2.87 × 10^−5^ day^−1^[15] & estimated
*λ*_*I*_12_*M*_1__	production rate of IL-12 by M1 macrophages	9.64 × 10^−2^ day^−1^[15] & estimated
*λ*_*I*_12_*M*_2_*i*_	production rate of IL-12 by M2i macrophages	9.64 × 10^−3^ day^−1^[13, 15] & estimated
*λ*_*I*_12_*D*_	production rate of IL-12 by DCs	9.64 × 10^−4^ day^−1^[13, 15] & estimated
*λ*_*T*_*α*_*M*2*i*_	production rate of TNF-*α* by M2 macrophage	1.07 × 10^−3^ day^−1^[13, 15] & estimated
*λ*_*T*_*α*_*M*1_	production rate of TNF-*α* by M1 macrophage	1.07 × 10^−1^ day^−1^[15] & estimated
*λ*_*I*_2_*T*_1__	production rate of IL-2 by Th1 cells	4.2 × 10^−4^ day^−1^[15] & estimated
*λ*_*GM*1_	production rate of GM-CSF by M1 macrophages	4.81 × 10^−3^ day^−1^[15] & estimated
*λ*_*GM*2_	production rate of GM-CSF by M2 macrophages	4.81 × 10^−4^ day^−1^[15] & estimated
*λ*_*Bi*_	production rate of bacteria	0.8 day^−1^[13]
*λ*_*I*_1*β*_*M*_2__	production rate of IL-1*β* by M2 macrophages	7.86 × 10^−4^ day^−1^ estimated
*λ*_*I*_1*β*_*M*_2*i*__	production rate of IL-1*β* by M2i macrophages	7.86 × 10^−3^ day^−1^ estimated
*λ*_*I*_6_*M*_2*i*__	production rate of IL-6 by M2i macrophages	1.74 × 10^−5^ day^−1^ estimated
*λ*_*I*_13_*T*_2__	production rate of IL-13 by Th2 cells	2.24 × 10^−4^ day^−1^[15, 16] & estimated
*λ*_*I*_13_*M*_2__	production rate of IL-13 by macrophages	5.94 × 10^−4^ day^−1^[15, 16] & estimated
*λ*_*I*_10_*M*2*i*_	production rate of IL-10 by M2i macrophages	6.67 × 10^−3^ day^−1^[13, 15] & estimated
*λ*_*I*_10_*M*2_	production rate of IL-10 by M2 macrophages	6.67 × 10^−4^ day^−1^[15] & estimated
*λ*_*I*_10_*D*_	production rate of IL-10 by DCs	10^−4^ day^−1^[13, 15] & estimated
*λ*_*I*_4_*T*_2__	production rate of IL-10 by Th2 cells	5.96 × 10^−4^ day^−1^[15, 57, 58] & estimated
*λ*_*I*_4_*M*_2__	production rate of IL-10 by M2 macrophages	2.38 × 10^−3^ day^−1^[15, 57, 58] & estimated
*λ*_*PM*_2*i*__	production rate of MCP-1 by M2i macrophages	4.86 × 10^−3^ day^−1^[15]
*λ*_*I*_*α*_*M*_2__	production rate of IFN-*α* by M2 macrophages	7.72 × 10^−8^ day^−1^ estimated
*λ*_*I*_*α*_*M*_2*i*__	production rate of IFN-*α* by M2i macrophages	7.72 × 10^−7^ day^−1^ estimated

**Table 3:** Parameters’ description and value.

Parameter	Description	Value
*d*_*M*1_	death rate of M1 macrophage	0.02 day^−1^[13]
*d*_*M*2_	death rate of M2 macrophage	0.008 day^−1^[13]
*d*_*M*2*i*_	death rate of infected M2 macrophage	0.02 day^−1^[13]
*d*_*T*_1__	death rate of Th1 cell	1.97 × 10^−1^ day^−1^[15, 59, 60]
*d*_*T*_2__	death rate of Th2 cell	1.97 × 10^−1^ day^−1^[15, 59, 60]
*d*_*D*_	death rate of dendritic cell	0.1 day^−1^[15]
*d*_*I*_*γ*__	degradation rate of IFN-*γ*	2.16 day^−1^[13]
*d*_*T*_*α*__	degradation rate of TNF-*α*	55.45 day^−1^[13, 15]
*d*_*I*_1*β*__	degradation rate of IL-1*β*	6.65 day^−1^[15]
*d*_*I*_2__	degradation rate of IL-2	2.376 day^−1^[15]
*d*_*I*_4__	degradation rate of IL-4	50 day^−1^[57, 61]
*d*_*I*_6__	degradation rate of IL-6	0.173 day^−1^[62]
*d*_*I*_10__	degradation rate of IL-10	8.32 day^−1^[63]
*d*_*I*_12__	degradation rate of IL-12	1.38 day^−1^[64]
*d*_*I*_13__	degradation rate of IL-13	12.47 day^−1^[15]
*d*_*G*_	degradation rate of GM-CSF	4.16 day^−1^[15]
*d*_*P*_	degradation rate of MCP-1	1.73 day^−1^[15]
*M*_0_	source of M1 macrophages	0.5 *g*/*cm*^3^ estimated
*T*_0_	Source of naive T cells	0.2 *g*/*cm*^3^ estimated
*D*_0_	inactive DC density	5 × 10^−2^ *g*/*cm*^3^[65]
*K*_*T*_1__	Th1 cell saturation	1 × 10^−1^ g/ml [66, 67]
*K*_*Be*_	bacterial saturation	2 × 10^−11^Â *gcm*^−3^[13] & estimated
*K*_*I*_*γ*__	IFN-*γ* saturation	2 × 10^−7^Â *gcm*^−3^[15]
*K*_*T*_*α*__	TNF-*α* saturation	1 × 10^−6^ g/*cm*^3^[15]
*K*_*G*_	GM-CSF saturation	1 × 10^−6^ g/*cm*^3^[15]
*K*_*I*_2__	IL-2 saturation	5 × 10^−7^ g/*cm*^3^[15]
*K*_*I*_4__	IL-4 saturation	2 × 10^−7^ g/*cm*^3^[15] & estimated
*K*_*I*_10__	IL-10 saturation	2 × 10^−7^ g/*cm*^3^[15]
*K*_*I*_12__	IL-12 saturation	1.5 × 10^−5^ g/*cm*^3^[15]
*K*_*I*_13__	IL-13 saturation	2 × 10^−7^ g/*cm*^3^[15]
*δ*	small parameter for numerical purpose	10^−4^[13] & estimated
*N*	burst size	50 [68]
*A*	source term of M2	0.05 g/*cm*^3^[15]
*K*_*M*_1__	M1 saturation	0.5 g/*cm*^3^[13]
*K*_*M*_2__	M2 saturation	1 g/*cm*^3^[13]
*K*_*I*_1_*β*_	IL-1*β* saturation	10^−8^ g/*cm*^3^ estimated
*χ*_*C*_	chemotaxis rate	10 g/*cm*^3^[13]

**Fig 3:**
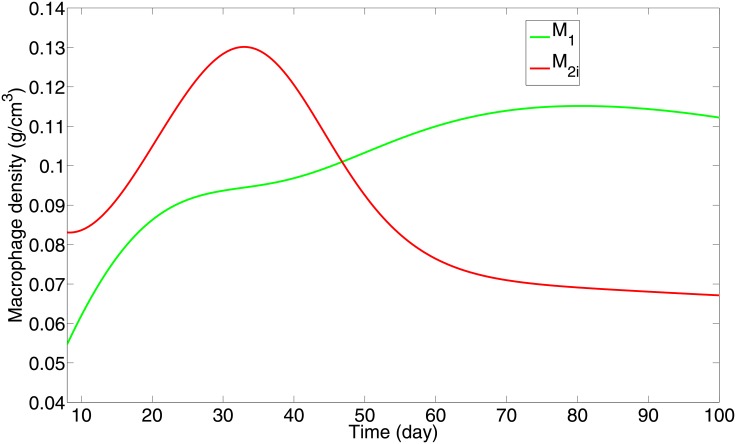
“Switching time” of macrophages; all the parameters and initial conditions are as in Fig 2. M1 macrophages become dominant over infected M2 macrophages at day 47.

As discussed in our previous work, [13], alveolar macrophages are specialized to recognize and eliminate most pathogens without causing inflammation. However when a strong inflammatory response is needed to eradicate pathogens, proinflammatory macrophages M1 migrate into the lung alveoli, and they eventually become dominant over the infected alveolar macrophages M2i. The time when this occurs we defined as the “switching time” [13]. From Fig 2, but more clearly from Fig 3, we see that, with the immune strength parameter *α* = 1, the switching time is around 47 days, which is in agreement with the typical switching time response established in Day et. al [13]. Thus *α* = 1 will be considered to be the parameter value of the immune strength for a normal healthy person. The switching time represents the balance between M1/M2i. On the other hand the parameter *α* represents a more comprehensive system that includes Th1 and Th2 cells and a larger network of cytokines. In this sense, the parameter *α* provides a more robust gauge of the immune strength and response of an individual to infection with Mtb.

Fig 2 shows that with initial bacterial load as in Eq (31), the granuloma continues to grow: R increases from 0.01 to 0.022 cm in 100 days. The densities of bacteria, Be and Bi, and the densities of M1 and Th1 increase while the densities of Th2 (after 10 days) and of M2, M2i decrease. We may conclude that the initial infection (Eq (31)) leads to a productive TB infection. The increase in *I*_*γ*_ and *I*_2_ in Fig 2 is a consequence of the increase in Th1, and the decrease in *I*_13_ and *I*_4_ is a consequence of decrease in *T*_2_ and *M*_2_. Similarly, the decrease in *I*_6_ and *P* follows the decrease in M2i. The fact that *G* is monotonically increasing means that M1 in Eq (20), which is increasing (in Fig 2) in time, dominates M2 which is decreasing in time.

If we take smaller initial values in Eq (31), the granuloma may decrease: for example, if *Be*(0) = 10^−6^ *g*/*cm*^3^, and *Bi*(0) = 10^−5^ *g*/*cm*^3^ then the radius decreases continuously from 0.01 cm to 0.0095 cm (not shown here).

As demonstrated experimentally in [48], there is great variability in the nature of non-human primate granulomas. For example, the number of bacteria may vary from 0 to 10^6^ CFU/granuloma [48]. In Fig 2, dthe average density of *B*_*i*_ approaches 0.027, which means (see the paragraph following Eq (30)) that the number of *B*_*i*_ in the granuloma at day 100 is $\frac{0.027}{10^{-7}}\times \frac{G(100)}{G(0)}=2.7\times 10^{5}\times \frac{G(100)}{G(0)}$ where *G*(*t*) is the volume of the granuloma at time *t*.

**Fig 4:**
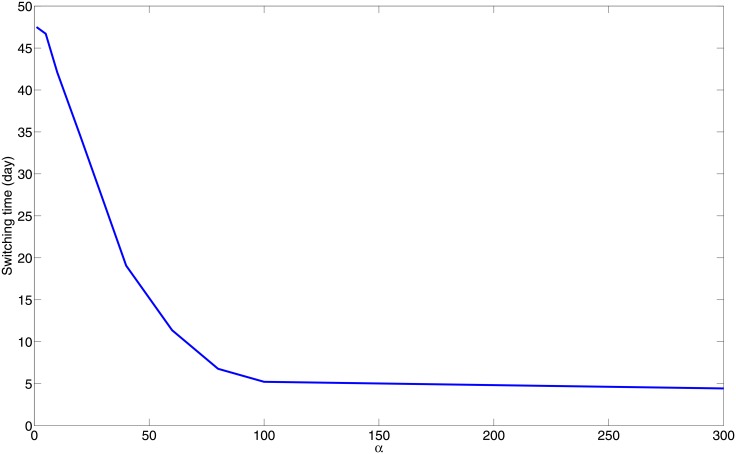
“Switching time” v.s. *α*; all the parameters (except *α*) and initial conditions are as in Fig 2. Since switching time cannot be shorter than at least two weeks [44], the biologically range of *α* is 0 < *α* < 50.

An individual with a stronger immune strength, that is, with a larger *α*, should have a shorter switching time, that is, the switching time should be a monotonically decreasing function of *α*. This is illustrated in Fig 4, which shows how the switching time decreases monotonically from 47 days to 4 days as the flux rate parameter *α* increases from 1 to 300. However, if the initial number of bacteria is increased or decreased, the corresponding profile of Fig 4 will slightly change (not shown here).

The switching time can range from a few weeks to two months [13]. From Fig 4, we see that under the initial conditions of Eq (30), the biologically relevant values of *α* lie in the interval 0 < *α* < 50.

**Fig 5:**
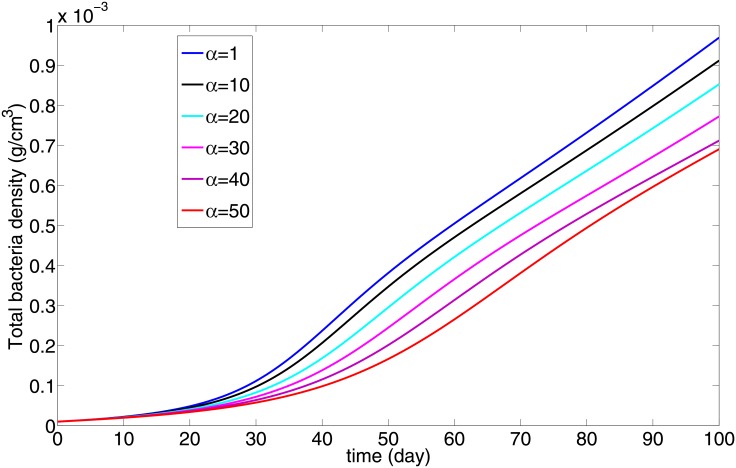
The total bacteria load (in *g*/*cm*^3^) v.s. *α*; all the parameters (except *α*) and initial conditions are the same as in Fig 2. The bacterial load decreases as the immune strength parameter *α* increases.

Fig 5 shows how the total number of bacteria grows in the first 100 days for individuals with different immune strength, as defined by the parameter *α*. We see that the total population of bacteria decreases as *α* increases.

**Fig 6:**
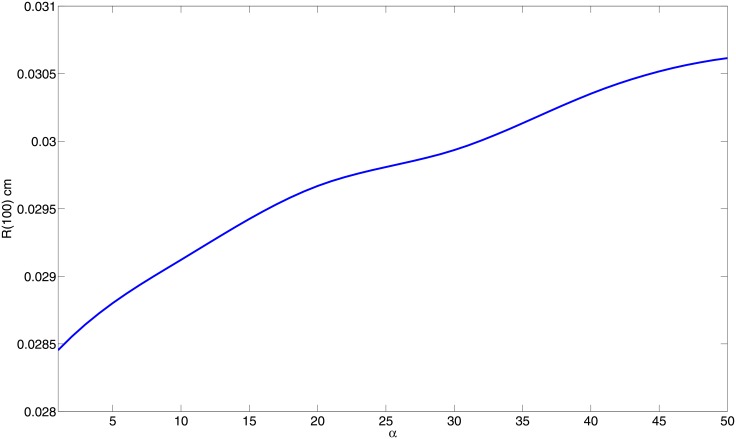
The radius of the granuloma at day 100 v.s. the immune strength parameter *α*; all the parameters (except *α*) and initial conditions are as in Fig 2. The radius at day 10 is monotonically increasing with respect to the parameter *α*.

Fig 6 profiles the radius of the granuloma at day 100, as a function of the parameter *α* in the biologically relevant range, where 0 < *α* < 50. As the immune response strength increases (i.e., as *α* increases) more immune cells arrive to, and will remain in, the granuloma, so *R*(100) will increase. While with increased *α* the total number of bacteria decreases (see Fig 5), the granuloma radius actually increases (see Fig 6), and hence there are increased inflammatory signals, and the resulting inflammation may damage the host tissue. We conclude that a very strong immune response to infection with Mtb is not necessarily beneficial.

We next use the model to determine efficacy of potential host-directed therapies for reducing the bacterial load. We begin with anti IL-10 Ab which is administered continuously (daily) for 30 days. We first assume that anti IL-10 reduces the production of IL-10 by 90%, i.e., the parameters *λ*_*I*_10_*M*_2*i*__, *λ*_*I*_10_*D*_ and *λ*_*I*_10_*M*_2__ are reduced by a factor 1/10 during the 30 days of drug treatment. We sought to determine the optimal time to begin treatment in order to maximize the reduction in total bacterial load (*B*_*e*_ and *B*_*i*_).

**Fig 7:**
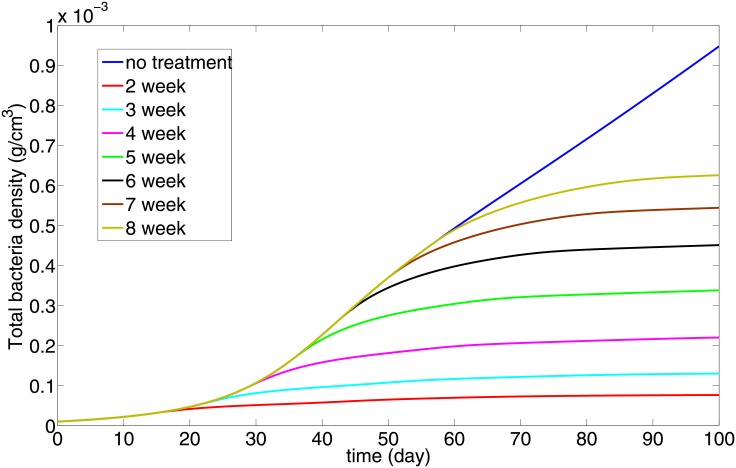
The total bacteria under IL-10 Ab treatment for *α* = 1; the treatment begins at different weeks and goes on for 30 days. The IL-10 Ab is assumed to reduce the production of IL-10 by 90%, i.e., the parameters *λ*_*I*_10_*M*_2*i*__, *λ*_*I*_10_*D*_ in Eq (14) and *λ*_*I*_10_*M*_2__ are reduced by factor 1/10 during the 30 days of the treatment. All the other parameter and initial values are the same as in Fig 2.

Fig 7 simulates the case *α* = 1 when anti IL-10 Ab is injected at the beginning of week *j*, where *j* = 2, 3, ⋯, 8. We see that there is a reduction in the bacterial load at day 100 when the Ab is administered as early as possible. Since most individuals infected with Mtb do not show symptoms for the first few weeks, what Fig 7 says is, simply, that treatment should start as soon as TB is diagnosed, for example, ideally early with skin test conversion.

**Fig 8:**
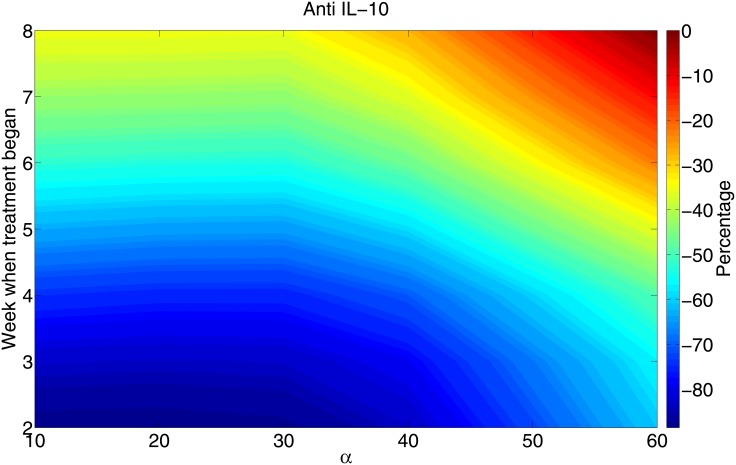
The percentage of total bacterial load reduction with anti IL-10 when the Ab is administered at different initial times (for 30 days) to patients with different immune strength *α*. The week when treatment begins is noted on the vertical axis. The color column indicates the percentage of bacterial reduction after 100 days from the initial time when the granuloma was formed. The parameter *λ*_*I*_10_*M*_2*i*__, *λ*_*I*_10_*D*_ and *λ*_*I*_10_*M*_2__ in Eq (14) are reduced by factor of 1/10, and all the other parameters (except *α*) and initial values are the same as in Fig 2.

Fig 8 shows the contour of the total bacteria load reduction percentage for general values of *α*. To create the percentage, we divided the *α* axis by 6 equidistant points, i.e, *α*_*i*_ = 10(1+*i*) (*i* = 0, ⋯, 5), and divided the treatment starting-week axis by 7 equidistant points, i.e., *w*_*j*_ = *j* (*j* = 2, ⋯, 8). For each pair (*α*_*i*_, *w*_*j*_), we computed the total bacteria, *B*(*α*_*i*_, *w*_*j*_), after 100 days, and determined the total bacteria load reduction percentage by the formula

$$\begin{array}{c} D_{ij}=\frac{B\left(\alpha_{i},w_{j}\right)-B_{0}}{B_{0}}, \end{array}$$

where *B*_0_ is the total bacteria concentration without treatment. The legend of Fig 8 scales the percentage of the total bacteria reduction (*B*_*i*_ and *B*_*e*_) with anti IL-10 Ab.

We see that delay in treatment always reduces its benefits in terms of reduction in the bacteria load. At the same time, if one patient has a stronger immune response than another patient, and both are treated with the same delay, the benefits in terms of the percent of bacteria reduction are less for a patient with a stronger immune response.

We assumed above that anti IL-10 Ab reduces IL-10 production by 90%. Results similar to Figs 7 and 8 can be established for different percentages of reductions (not shown here).

**Fig 9:**
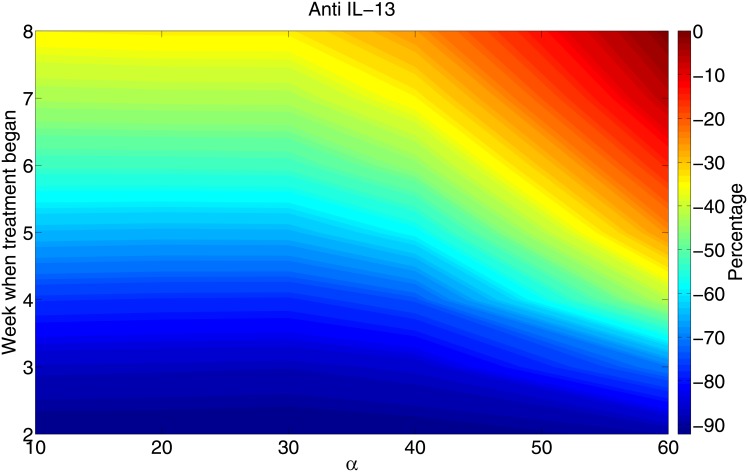
The percentage of total bacteria load reduction with anti IL-13 when the Ab is administered at different initial times (for 30 days) to patients with different ‘immune strength’ *α*. The parameter *λ*_*I*_13_*T*_2__ and *λ*_*I*_13_*M*_2__ in Eq (16) are reduced by factor of 1/10, and all the other parameters (except *α*) and initial values are the same as in Fig 2.

Fig 9 simulates the percent bacteria reduction using anti IL-13 Ab. The results are similar to those with anti IL-10 Ab.

## Conclusion

A granuloma is a structure consisting of a collection of immune cells that surrounds, isolates and quarantines pathogens. Granulomas are the histologic hallmark of infection with Mtb. The dynamics of a granuloma depend not only on the number of inhaled bacteria, but also on the immune response of the host, most importantly on the immune activities of macrophages and lymphocytes.

In the present work we developed a mathematical model of the dynamics of a granuloma by a system of PDEs. Although the process of growth or size reduction of a granuloma is probably similar in all granulomas, there could be differences in granulomas in different organs of the body. There may also be differences which depend on the type of Mtb. In this paper we focused on Mtb granulomas in the lung, where M2 macrophages are used to represent alveolar macrophages [13], but the model could be modified to describe granulomas in other parts of the body arising from infection with other Mtb and other intracellular pathogens of macrophages that form granulomas.

In previous work, Hao et al. [15] considered granulomas that arise in sarcoidosis (a disease with unknown etiology but suspected to be of mycobacterial origin). They developed a mathematical model that was validated by tissue data, and used this model to explore potential therapeutic agents. The mathematical model of the present paper shares some common features with the one introduced by Hao et. al. [15]. However it also has several important differences. The main new aspect is that for infection with Mtb we need to differentiate between resident alveolar macrophages (alternatively activated macrophages, or M2 macrophages) and pro-inflammatory macrophages (M1 macrophages, or classically activated macrophages) that arise with activation of the adaptive immune system. Furthermore, as a result of ingesting bacteria, some cytokines play different roles than they do in sarcoidosis.

The ‘strength’ of the adaptive immune response, in our model, is represented by a parameter *α*, the flux rate by which T cells and M1 macrophages that immigrated from the lymph nodes enter into the granuloma through its boundary. The parameter *α* is negatively correlated with the ‘switching time’ (ST) concept introduced in [13], namely, the time it takes for the number of M1 macrophages to surpass the number of infected alveolar (M2) macrophages. Indeed, extending the concept of ST from the ODE model in [13] to the present more comprehensive PDE model, we show that ST is a monotone decreasing function of *α*; the immune strength parameter *α* increases as the ST decreases (Fig 4).

Simulations of the model show how the bacterial load decreases and the radius of the granuloma increases as the immune strength increases (Figs 5 and 6). This implies that there is a risk associated with increased immune strength, since while the bacterial load is kept under control the inflammatory signals and the inflammation caused by excessive response of T cells may be harmful to the patients’ lung tissue. The model was used to determine the efficacy of potential host-directed therapies, such as anti IL-10 Ab and anti IL-13 Ab. We show that with the same dosing level for a 30 day period, delay in the start of the treatment always reduces the benefits in terms of the residual bacterial load (Fig 7). We also show that, under the same treatment, an individual with a stronger immune strength, *α*, will get greater benefits (Figs 8 and 9). This suggests that dosing regimen could be adjusted based on the patient’s immune system: An individual whose ‘adaptive immune strength’ is somehow known to be very strong will require a lower dose of the drug.

It is known [49, 50] that Mtb granulomas eventually undergo characteristic calcification, which has the same density as bone, making it visible on diagnostic X-rays. Calcification is generally assumed to occur in old granulomas [51]. Since we are concerned in this paper with granuloma formation during the first few months from initial infection, we did not include calcification effects in our model.

In developing the mathematical model we ignored some of the important populations of immune cells. For simplicity we did not include CD8+ T cells in the model whose role can be similar to that of Th1 cells, and B cells which also regulate immune responses. We also did not include Treg cells whose main function is to regulate T cell function in order to control inflammation. The role of Th-17 cells in human granuloma is not known [44], and they were also not included in the model. We also did not include neutrophils whose role as “double edge sword” needs further experimental evaluation [52]. Finally we did not include in our model the oxygen transport within a granuloma [53]. The present paper can be considered as a first step towards a more comprehensive and predictive model of the dynamics of granulomas in response to infection.

## Parameter values

Although the etiology of sarcoidosis remains unclear, there are similarities in the immune response between TB and sarcoidosis, at least during their initial phases [45, 46]. For this reason we have taken parameter values from sarcoidosis [15] whenever such parameters were not available for Mtb granulomas. We have also taken parameters from earlier work by Day et. al. [13] which dealt with Mtb, and from several experimental papers. There were still several parameters for which we were unable to find any values, and these were estimated as follows.

We assumed that the rate *M*_0_ → *M*_1_ is larger than the rate *M*_2_ → *M*_1_, taking *λ*_*M*_0__ larger than *λ*_*M*_1__. We also assumed that the source of M1 macrophage (M0) is larger than the source of naive T cells (T0), taking M0 = 0.5 *g*/*cm*^3^, and T0 = 0.2 *g*/*cm*^3^. The estimation of *λ*_*D*_ and *λ*_*T*_1_*M*_1__ and the fitting of the parameter *λ*_*M*_0__ were done in order to ensure that granulomas can have a steady state, i.e., that the radius of granuloma, *R*, which is increasing under the initial condition (31) will be decreasing if the initial bacterial load is significantly smaller.

### Sensitivity analysis

In the system of Eqs (1)–(20) there appear 36 production rates which were estimated in Table 2. This number is too large for sensitivity analysis. We therefore lumped together the production rates of each cytokine. For instance, in Eq (14) we shall vary the vector

$$\begin{array}{c} \vec{\lambda_{I_{10}}}=\left(\lambda_{I_{10}M_{2}},\lambda_{I_{10}D},\lambda_{I_{10}M_{2i}}\right) \end{array}$$

by the same factor for all of these components, i.e., we shall sample

$$\begin{array}{c} \theta_{I_{10}}\vec{\lambda_{I_{10}}}\;\text{for}\;\frac{1}{2}\leq \theta_{I_{10}}\leq 2 \end{array}$$

rather than each components of $\vec{\lambda_{I_{10}}}$ separately. We have 12 equations for the cytokines, Eqs (9)–(20), and hence 12 variables to be sampled,

$$\begin{array}{c} \theta_{P},\theta_{I_{1\beta }},\cdots ,\theta_{G}. \end{array}$$ (32)

We followed the sensitivity analysis method described in [54]. We performed Latin hypercube sampling and generated 100 samples to calculate the partial rank correlation coefficient (PRCC) and p-values with respect to

- the granuloma radius, *R*, at day t = 100;
- the total bacteria, *B*, at day t = 100.

**Fig 10:**
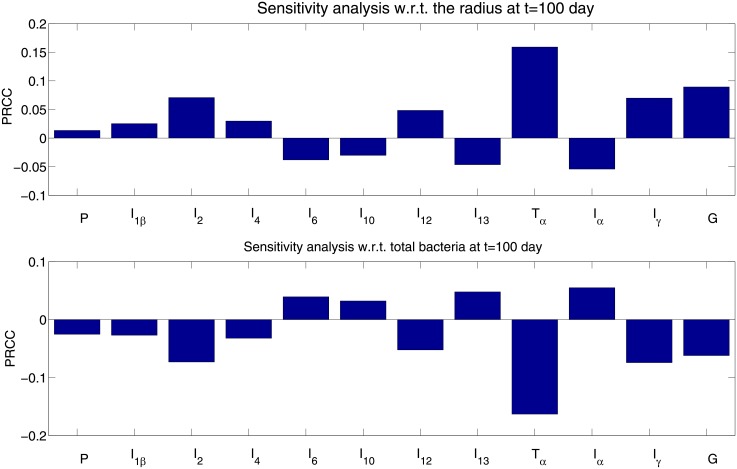
The sensitivity analysis for the cytokine production rates.

The results are shown in Fig 10, where, for simplicity, we replaced the variable Eq (32) by the corresponding cytokines,

$$\begin{array}{c} P,I_{1\beta },\cdots ,G. \end{array}$$

We see that *T*_*α*_ and *I*_*γ*_ are positively correlated with *R* and negatively correlated with *B*. Indeed, *T*_*α*_ and *I*_*γ*_ bring M1 cells into the granuloma (which increases R), and they also inhibit production of *B*_*i*_ (which decreases B). Similarly, *I*_2_ increases proliferation of Th1 cells and hence the production of *I*_*γ*_, so it is positively correlated with *R* and negatively correlated with B. On the other hand *I*_13_ blocks Th1 proliferation, and is negatively correlated with *R* and positively correlated with B. The other components in Fig 10 can similarly be explained.

From Fig 10 we conclude that any parameter that is positively (negatively) correlated with R is at the same time, negatively (positively) correlated with B.

We carried out the same sensitivity analysis with respect to the production parameters of the cells and found the same phenomenon, with the exception of the parameter *λ*_*M*_0__: *λ*_*M*_0__ is highly positively correlated with R and mildly positively correlated with B. This means that if more macrophages enter the granuloma, the granuloma will grow at a faster rate than the bacteria, so that the bacteria concentration will decrease.

## Computational method

In order to illustrate our numerical method, we consider the following convection-diffusion equation:

$$\begin{array}{c} \frac{\partial X}{\partial t}+div\left(vX\right)=D\nabla^{2}X+F_{X}, \end{array}$$ (33)

where *F*_*X*_ accounts for all the ‘active’ terms. Since the model we consider is a free boundary problem, we employ moving mesh method to compute it. Then Eq (33) can be written in the total derivative form

$$\begin{array}{c} \frac{dX(r(t),t)}{dt}+div\left(v\right)X=D\nabla^{2}X\left(r\left(t\right),t\right)+F_{X}. \end{array}$$

Let $r_{i}^{n}$, $X_{i}^{n}$ denote numerical approximations of i-th grid point and $X(r_{i}^{n},t)$ respectively when *t* = *nτ*, where *τ* is the time stepsize. The discretization is derived by the explicit Euler finite difference scheme, i.e.,

$$\begin{array}{c} \frac{X_{i}^{n+1}-X_{i}^{n}}{\tau }+\left(\frac{v_{r}}{r_{i}^{n}}+v_{i}^{n}\right)X_{i}^{n}=D\left(X_{rr}+\frac{X_{r}}{r_{i}^{n}}\right)+F_{X}, \end{array}$$

where $X_{r}=\frac{h_{-1}^{2}X_{i+1}^{n}-h_{1}^{2}X_{i-1}^{n}-\left(h_{1}^{2}-h_{-1}^{2}\right)X_{i}^{n}}{h_{1}\left(h_{-1}^{2}-h_{1}h_{-1}\right)}$, $X_{rr}=2\frac{h_{-1}X_{i+1}^{n}-h_{1}X_{i-1}^{n}+\left(h_{1}-h_{-1}\right)X_{i}^{n}}{h_{1}\left(h_{1}h_{-1}-h_{-1}^{2}\right)}$, $h_{-1}=r_{i-1}^{n}-r_{i}^{n}$ and $h_{1}=r_{i+1}^{n}-r_{i}^{n}$. Then the mesh is moving by $r_{i}^{n+1}=r_{i}^{n}+v_{i}^{n}\tau$, where $v_{i}^{n}$ is solved by the velocity equation. In order to make the Euler method stable, we take $\tau \leq \frac{min\{h_{1},h_{-1}\}}{2D}$.

## References

[1] Guirado E, Schlesinger LS. Modeling the Mycobacterium tuberculosis Granuloma—the Critical Battlefield in Host Immunity and Disease. Front Immunol. 2013;4:98 10.3389/fimmu.2013.00098 23626591 PMC3631743

[2] Orme IM, Basaraba RJ. The formation of the granuloma in tuberculosis infection. Semin Immunol. 2014;26(6):601–609. 10.1016/j.smim.2014.09.009 25453231

[3] Guirado E, Schlesinger LS, Kaplan G. Macrophages in tuberculosis: friend or foe. Semin Immunopathol. 2013;35(5):563–583. 10.1007/s00281-013-0388-2 23864058 PMC3763202

[4] Reyes N, Bettin A, Reyes I, Geliebter J. Microarray analysis of the in vitro granulomatous response to Mycobacterium tuberculosis H37Ra. Colomb Med. 2015;46(1):26–32. 26019382 PMC4437284

[5] Silva Miranda M, Breiman A, Allain S, Deknuydt F, Altare F. The tuberculous granuloma: an unsuccessful host defence mechanism providing a safety shelter for the bacteria? Clin Dev Immunol. 2012;2012:139127 10.1155/2012/139127 22811737 PMC3395138

[6] Shaler CR, Horvath CN, Jeyanathan M, Xing Z. Within the Enemy’s Camp: contribution of the granuloma to the dissemination, persistence and transmission of Mycobacterium tuberculosis. Front Immunol. 2013;4:30 10.3389/fimmu.2013.00030 23420646 PMC3572501

[7] Lin PL, Flynn JL. Understanding latent tuberculosis: a moving target. J Immunol. 2010;185(1):15–22. 10.4049/jimmunol.0903856 20562268 PMC3311959

[8] Ehlers S, Schaible UE. The granuloma in tuberculosis: dynamics of a host-pathogen collusion. Front Immunol. 2012;3:411 10.3389/fimmu.2012.00411 23308075 PMC3538277

[9] Lin Y, Gong J, Zhang M, Xue W, Barnes PF. Production of monocyte chemoattractant protein 1 in tuberculosis patients. Infect Immun. 1998;66(5):2319–2322. 9573123 10.1128/iai.66.5.2319-2322.1998 PMC108197

[10] Chroneos ZC, Jagannath C. Immunoregulatory Role of GM-CSF in Pulmonary Tuberculosis. Understanding Tuberculosis—Analyzing the Origin of Mycobacterium Tuberculosis Pathogenicity. 2012;.

[11] Venkayya R, Lam M, Willkom M, Grunig G, Corry DB, Erle DJ. The Th2 lymphocyte products IL-4 and IL-13 rapidly induce airway hyperresponsiveness through direct effects on resident airway cells. Am J Respir Cell Mol Biol. 2002;26(2):202–208. 10.1165/ajrcmb.26.2.4600 11804871

[12] Veremeyko T, Siddiqui S, Sotnikov I, Yung A, Ponomarev ED. IL-4/IL-13-dependent and independent expression of miR-124 and its contribution to M2 phenotype of monocytic cells in normal conditions and during allergic inflammation. PLoS ONE. 2013;8(12):e81774 10.1371/journal.pone.0081774 24358127 PMC3864800

[13] Day J, Friedman A, Schlesinger LS. Modeling the immune rheostat of macrophages in the lung in response to infection. Proc Natl Acad Sci USA. 2009;106(27):11246–11251. 10.1073/pnas.0904846106 19549875 PMC2708732

[14] Mayer-Barber KD, Andrade BB, Oland SD, Amaral EP, Barber DL. Host-directed therapy of tuberculosis based on interleukin-1 and type I interferon crosstalk. Nature. 2014;511(7507):99–103. 10.1038/nature13489 24990750 PMC4809146

[15] Hao W, Crouser ED, Friedman A. Mathematical model of sarcoidosis. Proc Natl Acad Sci USA. 2014;111(45):16065–16070. 10.1073/pnas.1417789111 25349384 PMC4234561

[16] Hancock A, Armstrong L, Gama R, Millar A. Production of interleukin 13 by alveolar macrophages from normal and fibrotic lung. Am J Respir Cell Mol Biol. 1998;18(1):60–65. 10.1165/ajrcmb.18.1.2627 9448046

[17] Janeway CA, Travers P, Walport M, Shlomchik MJ. Immunobiology, 5th edition, The Immune System in Health and Disease; 2001.

[18] Yates A, Callard R, Stark J. Combining cytokine signalling with T-bet and GATA-3 regulation in Th1 and Th2 differentiation: a model for cellular decision-making. J Theor Biol. 2004;231(2):181–196. 10.1016/j.jtbi.2004.06.013 15380383

[19] Gammack D, Doering CR, Kirschner DE. Macrophage response to Mycobacterium tuberculosis infection. J Math Biol. 2004;48(2):218–242. 10.1007/s00285-003-0232-8 14745511

[20] Baumgart DC, Carding SR. Inflammatory bowel disease: cause and immunobiology. Lancet. 2007;369(9573):1627–1640. 10.1016/S0140-6736(07)60750-8 17499605

[21] Maloy KJ, Powrie F. Intestinal homeostasis and its breakdown in inflammatory bowel disease. Nature. 2011;474(7351):298–306. 10.1038/nature10208 21677746

[22] Jayaraman P, Sada-Ovalle I, Nishimura T, Anderson AC, Kuchroo VK, Remold HG, et al IL-1beta promotes antimicrobial immunity in macrophages by regulating TNFR signaling and caspase-3 activation. J Immunol. 2013;190(8):4196–4204. 10.4049/jimmunol.1202688 23487424 PMC3622150

[23] Buttner C, Skupin A, Reimann T, Rieber EP, Unteregger G, Geyer P, et al Local production of interleukin-4 during radiation-induced pneumonitis and pulmonary fibrosis in rats: macrophages as a prominent source of interleukin-4. Am J Respir Cell Mol Biol. 1997;17(3):315–325. 10.1165/ajrcmb.17.3.2279 9308918

[24] Yang CT, Cambier CJ, Davis JM, Hall CJ, Crosier PS, Ramakrishnan L. Neutrophils exert protection in the early tuberculous granuloma by oxidative killing of mycobacteria phagocytosed from infected macrophages. Cell Host Microbe. 2012;12(3):301–312. 10.1016/j.chom.2012.07.009 22980327 PMC3638950

[25] Eruslanov EB, Lyadova IV, Kondratieva TK, Majorov KB, Scheglov IV, Orlova MO, et al Neutrophil responses to Mycobacterium tuberculosis infection in genetically susceptible and resistant mice. Infect Immun. 2005;73(3):1744–1753. 10.1128/IAI.73.3.1744-1753.2005 15731075 PMC1064912

[26] Gammack D, Ganguli S, Marino S, Segovia-Juarez J, Kirschner DE. Understanding the immune response in tuberculosis using different mathematical models and biological scales. SIAM Mult Mode Simu. 2005;3(2):312–345. 10.1137/040603127

[27] Marino S, Myers A, Flynn JL, Kirschner DE. TNF and IL-10 are major factors in modulation of the phagocytic cell environment in lung and lymph node in tuberculosis: a next-generation two-compartmental model. J Theor Biol. 2010;265(4):586–598. 10.1016/j.jtbi.2010.05.012 20510249 PMC3150786

[28] Cilfone NA, Perry CR, Kirschner DE, Linderman JJ. Multi-scale modeling predicts a balance of tumor necrosis factor-alpha and interleukin-10 controls the granuloma environment during Mycobacterium tuberculosis infection. PLoS ONE. 2013;8(7):e68680 10.1371/journal.pone.0068680 23869227 PMC3711807

[29] Fallahi-Sichani M, El-Kebir M, Marino S, Kirschner DE, Linderman JJ. Multiscale computational modeling reveals a critical role for TNF-alpha receptor 1 dynamics in tuberculosis granuloma formation. J Immunol. 2011;186(6):3472–3483. 10.4049/jimmunol.1003299 21321109 PMC3127549

[30] Marino S, Cilfone NA, Mattila JT, Linderman JJ, Flynn JL, Kirschner DE. Macrophage polarization drives granuloma outcome during Mycobacterium tuberculosis infection. Infect Immun. 2015;83(1):324–338. 10.1128/IAI.02494-14 25368116 PMC4288886

[31] Wigginton JE, Kirschner D. A model to predict cell-mediated immune regulatory mechanisms during human infection with Mycobacterium tuberculosis. J Immunol. 2001;166(3):1951–1967. 10.4049/jimmunol.166.3.1951 11160244

[32] Cilfone NA, Ford CB, Marino S, Mattila JT, Gideon HP, Kirschner DE, et al Computational modeling predicts IL-10 control of lesion sterilization by balancing early host immunity-mediated antimicrobial responses with caseation during mycobacterium tuberculosis infection. J Immunol. 2015;194(2):664–677. 10.4049/jimmunol.1400734 25512604 PMC4283220

[33] Linderman JJ, Cilfone NA, Pienaar E, Gong C, Kirschner DE. A multi-scale approach to designing therapeutics for tuberculosis. Integr Biol (Camb). 2015;7(5):591–609. 10.1039/C4IB00295D 25924949 PMC4436084

[34] Pienaar E, Cilfone NA, Lin PL, Dartois V, Mattila JT, Flynn JL, et al A computational tool integrating host immunity with antibiotic dynamics to study tuberculosis treatment. J Theor Biol. 2015;367:166–179. 10.1016/j.jtbi.2014.11.021 25497475 PMC4332617

[35] Stanley SA, Johndrow JE, Manzanillo P, Cox JS. The Type I IFN response to infection with Mycobacterium tuberculosis requires ESX-1-mediated secretion and contributes to pathogenesis. J Immunol. 2007;178(5):3143–3152. 10.4049/jimmunol.178.5.3143 17312162

[36] Deepak P, Kumar S, Kishore D, Acharya A. IL-13 from Th2-type cells suppresses induction of antigen-specific Th1 immunity in a T-cell lymphoma. Int Immunol. 2010;22(1):53–63. 10.1093/intimm/dxp114 19951958

[37] Okada M, Kita Y, Kanamaru N, Hashimoto S, Uchiyama Y. Anti-IL-6 receptor antibody causes less promotion of tuberculosis infection than anti-TNF- antibody in mice. Clin Dev Immunol. 2011;2011:404929 10.1155/2011/404929 21603208 PMC3095415

[38] Ma J, Yang B, Yu S, Zhang Y, Zhang X. Tuberculosis antigen-induced expression of IFN-gamma in tuberculosis patients inhibits production of IL-1beta. FASEB J. 2014;28(7):3238–3248. 10.1096/fj.13-247056 24675363

[39] Pouliot P, Turmel V, Gelinas E, Laviolette M, Bissonnette EY. Interleukin-4 production by human alveolar macrophages. Clin Exp Allergy. 2005;35(6):804–810. 10.1111/j.1365-2222.2005.02246.x 15969673

[40] Nagabhushanam V, Solache A, Ting LM, Escaron CJ, Zhang JY. Innate inhibition of adaptive immunity: Mycobacterium tuberculosis-induced IL-6 inhibits macrophage responses to IFN-gamma. J Immunol. 2003;171(9):4750–4757. 10.4049/jimmunol.171.9.4750 14568951

[41] VanHeyningen TK, Collins HL, Russell DG. IL-6 produced by macrophages infected with Mycobacterium species suppresses T cell responses. J Immunol. 1997;158(1):330–337. 8977207

[42] Bona CA, Revillard JP. Cytokines and Cytokine Receptors: Physiology and Pathological Disorders. CRC Press 2001;.

[43] Oswald IP, Wynn TA, Sher A, James SL. Interleukin 10 inhibits macrophage microbicidal activity by blocking the endogenous production of tumor necrosis factor alpha required as a costimulatory factor for interferon gamma-induced activation. Proc Natl Acad Sci USA. 1992;89(18):8676–8680. 10.1073/pnas.89.18.8676 1528880 PMC49983

[44] Flynn JL, Chan J, Lin PL. Macrophages and control of granulomatous inflammation in tuberculosis. Mucosal Immunol. 2011;4(3):271–278. 10.1038/mi.2011.14 21430653 PMC3311958

[45] Calaras D, Munteanu O, Botnaru V. Sarcoidosis and tuberculosis: A rare combination? Euro Resp J. 2012;.

[46] Calaras D, Munteanu O, Botnaru V. Overview of Sarcoidosis. www.healthcommunities.com. 2015;.

[47] Gil O, Diaz I, Vilaplana C, Tapia G, Diaz J, et al Granuloma encapsulation is a key factor for containing tuberculosis infection in minipigs. PLoS ONE. 2010;5(4):e10030 10.1371/journal.pone.0010030 20386605 PMC2850319

[48] Gideon HP, Phuah J, Myers AJ, Bryson BD, Rodgers MA, Coleman MT, et al Variability in tuberculosis granuloma T cell responses exists, but a balance of pro- and anti-inflammatory cytokines is associated with sterilization. PLoS Pathog. 2015;11(1):e1004603 10.1371/journal.ppat.1004603 25611466 PMC4303275

[49] Co DO, Hogan LH, Kim SI, Sandor M. Mycobacterial granulomas: keys to a long-lasting host-pathogen relationship. Clin Immunol. 2004;113(2):130–136. 10.1016/j.clim.2004.08.012 15451467

[50] Steckelberg JM. Granuloma: What does it mean? http://www.mayoclinic.org/. 2015;.

[51] Medicinenet. Definition of Granuloma. http://www.medicinenet.com/. 2015;.

[52] Cardona PJ. Understanding tuberculosis-analyzing the orgin of mycobacterium tuberculosis pathogenicity. InTech. 2012;.

[53] Datta M, Via LE, Chen W, Baish JW, Xu L, Barry CE, et al Mathematical Model of Oxygen Transport in Tuberculosis Granulomas. Ann Biomed Eng. 2015;. 10.1007/s10439-015-1415-3 26253038 PMC4795989

[54] Marino S, Hogue IB, Ray CJ, Kirschner DE. A methodology for performing global uncertainty and sensitivity analysis in systems biology. J Theor Biol. 2008;254(1):178–196. 10.1016/j.jtbi.2008.04.011 18572196 PMC2570191

[55] Hao W, Friedman A. The LDL-HDL profile determines the risk of atherosclerosis: a mathematical model. PLoS ONE. 2014;9(3):e90497 10.1371/journal.pone.0090497 24621857 PMC3951264

[56] Friedman A, Hao W. A mathematical model of atherosclerosis with reverse cholesterol transport and associated risk factors. Bull Math Biol. 2015;77(5):758–781. 10.1007/s11538-014-0010-3 25205457

[57] Lo W, I AR, Friedman A. Inflammatory Bowel Disease. Preprint. 2015;.

[58] Liang HE, Reinhardt RL, Bando JK, Sullivan BM, Ho IC, Locksley RM. Divergent expression patterns of IL-4 and IL-13 define unique functions in allergic immunity. Nat Immunol. 2012;13(1):58–66. 10.1038/ni.2182 PMC3242938 22138715

[59] Hao W, Rovin BH, Friedman A. Mathematical model of renal interstitial fibrosis. Proc Natl Acad Sci USA. 2014;111(39):14193–14198. 10.1073/pnas.1413970111 25225370 PMC4191765

[60] Hao W, Marsh C, Friedman A. A Mathematical Model of Idiopathic Pulmonary Fibrosis. PLoS ONE. 2015;10(9):e0135097 10.1371/journal.pone.0135097 26348490 PMC4562674

[61] Conlon PJ, Tyler S, Grabstein KH, Morrissey P. Interleukin-4 (B-cell stimulatory factor-1) augments the in vivo generation of cytotoxic cells in immunosuppressed animals. Biotechnol Ther. 1989;1(1):31–41. 2562642

[62] Lu ZY, Brailly H, Wijdenes J, Bataille R, Rossi JF. Measurement of whole body interleukin-6 (IL-6) production: prediction of the efficacy of anti-IL-6 treatments. Blood. 1995;86(8):3123–3131. 7579407

[63] Li L, Elliott JF, Mosmann TR. IL-10 inhibits cytokine production, vascular leakage, and swelling during T helper 1 cell-induced delayed-type hypersensitivity. J Immunol. 1994;153(9):3967–3978. 7930605

[64] Bajetta E, Del Vecchio M, Mortarini R, Nadeau R, Rakhit A. Pilot study of subcutaneous recombinant human interleukin 12 in metastatic melanoma. Clin Cancer Res. 1998;4(1):75–85. 9516955

[65] Haller Hasskamp J, Zapas JL, Elias EG. Dendritic cell counts in the peripheral blood of healthy adults. Am J Hematol. 2005;78(4):314–315. 10.1002/ajh.20296 15795906

[66] Cosio MG, Majo J, Cosio MG. Inflammation of the airways and lung parenchyma in COPD: role of T cells. Chest. 2002;121(5 Suppl):160S–165S. 10.1378/chest.121.5_suppl.160S 12010846

[67] Purwar R, Campbell J, Murphy G, Richards WG, Clark RA, Kupper TS. Resident memory T cells (T(RM)) are abundant in human lung: diversity, function, and antigen specificity. PLoS ONE. 2011;6(1):e16245 10.1371/journal.pone.0016245 21298112 PMC3027667

[68] Repasy T, Lee J, Marino S, Martinez N, Kirschner DE. Intracellular bacillary burden reflects a burst size for Mycobacterium tuberculosis in vivo. PLoS Pathog. 2013;9(2):e1003190 10.1371/journal.ppat.1003190 23436998 PMC3578792

